# Etiologia da Doença Pericárdica: Quem Procura Acha!

**DOI:** 10.36660/abc.20230704

**Published:** 2023-11-14

**Authors:** Alexandre Siciliano Colafranceschi, Sofia Vega Colafranceschi

**Affiliations:** 1 Instituto Nacional de Cardiologia Rio de Janeiro RJ Brasil Instituto Nacional de Cardiologia – Cirurgia Cardíaca, Rio de Janeiro, RJ – Brasil; 2 Estácio de Sá Higher Education Society Rio de Janeiro RJ Brasil Estácio de Sá Higher Education Society, Rio de Janeiro, RJ – Brasil

**Keywords:** Pericárdio, Doenças Cardiovasculares, Diagnóstico

O saco pericárdico consiste em camadas fibroelásticas, conhecidas como camadas visceral e parietal, separadas pela cavidade pericárdica. Essa cavidade normalmente contém 15 a 50 ml de ultrafiltrado derivado de plasma em indivíduos saudáveis. As doenças pericárdicas são relativamente comuns na prática clínica, apresentando-se isoladamente ou como parte de distúrbios sistêmicos.

As causas dessas doenças variam e são complexas, mas o pericárdio normalmente responde com inflamação de suas camadas e potencial aumento da produção de líquido pericárdico. A inflamação persistente pode levar a um pericárdio endurecido e calcificado, muitas vezes espessado, com possível progressão para constrição pericárdica. Em alguns casos, a inflamação pericárdica aguda domina a apresentação clínica, tornando o excesso de líquido pericárdico menos relevante. Em contrapartida, em outros casos, o acúmulo de líquido e suas consequências clínicas, como tamponamento cardíaco e pericardite constritiva, ocupam o centro das atenções. Anomalias congênitas como ausência de pericárdio e cistos pericárdicos são geralmente raras e assintomáticas. Apesar da natureza não essencial do pericárdio para a função cardíaca normal, o pericárdio doente, apresentando-se como pericardite aguda ou recorrente, derrame pericárdico, tamponamento cardíaco e constrição pericárdica, pode representar desafios significativos no tratamento e até mesmo colocar a vida em risco.

Em contraste com doença arterial coronariana, insuficiência cardíaca, doença valvar e outros tópicos da área de cardiologia, existem poucos dados de ensaios randomizados para orientar os médicos no manejo das doenças pericárdicas. Embora não existam diretrizes da American Heart Association/American College of Cardiology sobre esse tema, a Sociedade Europeia de Cardiologia^1^ e a Sociedade Brasileira de Cardiologia^2^ publicaram diretrizes úteis, ainda que tenham dez anos, para o diagnóstico e manejo de doenças pericárdicas.

Determinar a causa da doença pericárdica é muitas vezes um desafio e muitos casos permanecem idiopáticos. No entanto, microrganismos, incluindo vírus e bactérias, condições sistémicas como neoplasias, doenças autoimunes, doenças do tecido conjuntivo, insuficiência renal, cirurgias cardíacas anteriores, infartos do miocárdio anteriores, trauma, dissecção aórtica, exposição à radiação e, raramente, medicamentos, foram todos associados a doenças pericárdicas.

Os médicos frequentemente enfrentam diversas questões diagnósticas e de manejo relacionadas às síndromes pericárdicas. As questões podem girar em torno de critérios diagnósticos, escolha de ferramentas diagnósticas, necessidade de hospitalização, viabilidade do manejo ambulatorial, estratégias médicas mais baseadas em evidências, momento do uso de corticosteroides e consideração de pericardiotomia cirúrgica. Uma questão persistente diz respeito à utilidade diagnóstica da biópsia pericárdica.

O estudo publicado nesta edição dos Arquivos Brasileiros de Cardiologia, Giuliani et al.^3^ examinaram retrospectivamente dados de 80 pacientes submetidos à biópsia pericárdica parietal entre 2011 e 2020 para avaliar o valor da biópsia pericárdica não guiada no estabelecimento de um diagnóstico etiológico e na orientação do manejo da doença pericárdica. As biópsias foram realizadas durante janelas pericárdicas terapêuticas por meio de diversas abordagens, incluindo subxifoide, videotoracoscopia ou toracotomia sob anestesia geral. Surpreendentemente, apenas 13,7% de todas as biópsias pericárdicas produziram um diagnóstico histopatológico conclusivo. Parece que os autores confiaram apenas na técnica de coloração com hematoxilina e eosina para análise histopatológica (coloração H&E), embora isso não esteja explicitamente declarado.^3^

A etiologia dos derrames pericárdicos permanece indeterminada em muitos casos, principalmente porque todo o espectro de métodos diagnósticos disponíveis é subutilizado em numerosas instituições. Esses métodos abrangem citologia (incluindo imunocitoquímica), histologia (incluindo imuno-histoquímica) e técnicas de biologia molecular (PCR para agentes microbianos cardiotrópicos). Além disso, a aplicação da pericardioscopia, das biópsias direcionadas do pericárdio e do epicárdio e as subsequentes análises teciduais melhoraram inquestionavelmente a nossa compreensão da fisiopatologia da doença pericárdica. A pericardioscopia permite o exame macroscópico do coração pulsante e de suas alterações macroscópicas relacionadas à doença. Também facilita a biópsia tecidual segura e precisa para investigação adicional.^4^

Quando todos esses métodos são empregados em pacientes com derrame pericárdico, o diagnóstico de derrame pericárdico “idiopático” torna-se obsoleto. Por exemplo, derrames pericárdicos autorreativos e linfocíticos são os diagnósticos mais prevalentes, representando 35% dos casos em um registro prospectivo de Marburg, seguidos por derrames malignos em 28%. Material genético viral foi detectado em fluidos e biópsias epicárdicas e pericárdicas em 12% dos casos, seguido por derrames pós-traumáticos/iatrogênicos em 15% e derrames purulentos/bacterianos em apenas 2%. Além do diagnóstico etiológico, abordagens terapêuticas podem ser escolhidas de acordo com a etiologia específica. Por exemplo, derrames autorreativos podem se beneficiar da instilação intrapericárdica de triancinolona, enquanto derrames neoplásicos podem responder à cisplatina ou tiotepa. Essa abordagem reduz efetivamente a recorrência do derrame pericárdico.^4^

Em conclusão, uma abordagem diagnóstica abrangente para derrames pericárdicos, combinada com pericardioscopia para amostragem de tecido direcionado, constitui a base para o tratamento intrapericárdico e sistêmico orientado pela etiologia, possivelmente melhorando os resultados e o prognóstico dos pacientes. Esta técnica, no entanto, é bastante exigente e só pode ser realizada num número limitado de centros de referência terciários experientes. Permite a aquisição segura de tecidos em doenças pericárdicas de origem desconhecida. Se o centro de atendimento tiver capacidade e capacidade apenas para realizar uma biópsia pericárdica não guiada e depender apenas da coloração H&E, o paciente não deve ser submetido a uma biópsia pericárdica para fins etiológicos, mas por necessidade terapêutica, como Giuliani, G. et al.^3^ mostrou que apenas 13,7% de todas as biópsias pericárdicas produziram um diagnóstico histopatológico conclusivo.
