# Supplementary material for: Etiologia da Doença Pericárdica: Quem Procura Acha!
Source: Arq Bras Cardiol. 2023 Nov 14;120(10):e20230704. [Article in Portuguese] doi: 10.36660/abc.20230704 (PMC10697687; doi:10.36660/abc.20230704)
Supplement: Translation

# Translation: Etiology of Pericardial Disease – Seek It, or You Shall not Find It!

**Authors:** Alexandre Siciliano Colafranceschi, Sofia Vega Colafranceschi

**Affiliations:** 1 Brazil Instituto Nacional de Cardiologia – Cirurgia Cardíaca, Rio de Janeiro, RJ – Brazil; 2 Brazil Estácio de Sá Higher Education Society, Rio de Janeiro, RJ – Brazil

**Keywords:** Pericardium, Cardiovascular Diseases, Diagnosis

The pericardial sac consists of fibroelastic layers, known as the visceral and parietal layers, separated by the pericardial cavity. This cavity typically contains 15 to 50 ml of a plasma-derived ultrafiltrate in healthy individuals. Pericardial diseases are relatively common in clinical practice, presenting either in isolation or as a part of systemic disorders.

The causes of these diseases vary and are intricate, but the pericardium typically responds with inflammation of its layers and potential increased production of pericardial fluid. Persistent inflammation may lead to a stiffened and calcified pericardium, often thickened, with possible progression to pericardial constriction. In some instances, acute pericardial inflammation dominates the clinical presentation, rendering excess pericardial fluid less relevant. In contrast, in other cases, the accumulation of fluid and its clinical consequences, such as cardiac tamponade and constrictive pericarditis, take center stage. Congenital abnormalities like the absence of the pericardium and pericardial cysts are generally rare and asymptomatic. Despite the non-essential nature of the pericardium for normal cardiac function, diseased pericardium, presenting as acute or recurrent pericarditis, pericardial effusion, cardiac tamponade, and pericardial constriction, can pose significant challenges in management and even become life-threatening.

In contrast to coronary artery disease, heart failure, valvular disease, and other topics in the field of cardiology, there are few data from randomized trials to guide physicians in the management of pericardial diseases. Although no American Heart Association/American College of Cardiology guidelines exist on this topic, the European Society of Cardiology^1^and the *Sociedade Brasileira de Cardiologia* ^2^have published useful guidelines, although they are ten years old, for the diagnosis and management of pericardial diseases.

Determining the cause of pericardial disease is often challenging, and many cases remain idiopathic. However, microorganisms, including viruses and bacteria, systemic conditions like neoplasia, autoimmune disorders, connective tissue diseases, renal failure, prior cardiac surgeries, previous myocardial infarctions, trauma, aortic dissection, radiation exposure, and rarely, drugs have all been linked to pericardial diseases.

Clinicians frequently grapple with various diagnostic and management queries related to pericardial syndromes. Questions may revolve around diagnostic criteria, the choice of diagnostic tools, hospitalization necessity, outpatient management feasibility, the most evidence-based medical strategies, the timing of corticosteroid use, and the consideration of surgical pericardiectomy. One persistent question pertains to the diagnostic utility of pericardial biopsy.

The study published in this *Arquivos Brasileiros de Cardiologia* issue, Giuliani et al.^3^retrospectively examined data from 80 patients who underwent parietal pericardial biopsy between 2011 and 2020 to assess the value of non-guided pericardial biopsy in establishing an etiological diagnosis and guiding pericardial disease management. The biopsies were performed during therapeutic pericardial windows via various approaches, including subxiphoid, video thoracoscopy, or thoracotomy under general anesthesia. Astonishingly, only 13.7% of all pericardial biopsies yielded a conclusive histopathological diagnosis. It appears that the authors solely relied on the hematoxylin and eosin staining technique for histopathological analysis (H&E stain), although this is not explicitly stated.^3^

The etiology of pericardial effusions remains undetermined in many cases, primarily because the full spectrum of available diagnostic methods is underutilized in numerous institutions. These methods encompass cytology (including immunocytochemistry), histology (including immunohistochemistry), and molecular biology techniques (PCR for cardiotropic microbial agents). Furthermore, applying pericardioscopy, targeted pericardial and epicardial biopsies and the subsequent tissue analyses have unquestionably enhanced our comprehension of pericardial disease pathophysiology. Pericardioscopy allows for the macroscopic examination of the pulsating heart and its disease-related macroscopic alterations. It also facilitates safe and precise tissue biopsy for further investigation.^4^

When all these methods are employed in patients with pericardial effusions, diagnosing “idiopathic” pericardial effusion becomes obsolete. For instance, autoreactive and lymphocytic pericardial effusions are the most prevalent diagnoses, accounting for 35% of cases in a prospective Marburg registry, followed by malignant effusions in 28%. Viral genetic material was detected in fluid and epi- and pericardial biopsies in 12% of cases, followed by post-traumatic/iatrogenic effusions in 15% and purulent/bacterial effusions in only 2%. Beyond the etiological diagnostics, therapeutic approaches can be chosen tailored to the specific etiology. For instance, autoreactive effusions may benefit from intrapericardial instillation of triamcinolone, while neoplastic effusions may respond to cisplatin or thiotepa. This approach effectively reduces the recurrence of pericardial effusion.^4^

In conclusion, a comprehensive diagnostic approach to pericardial effusions, combined with pericardioscopy for targeted tissue sampling, forms the basis for etiology-driven intrapericardial and systemic treatment, possibly improving patient outcomes and prognosis. This technique, however, is quite demanding and can be performed only in a limited number of experienced tertiary referral centers. It permits safe tissue acquisition in pericardial diseases of unknown origin. If the care delivery center only has the ability and capability to perform a non-guided pericardial biopsy and relies only on H&E staining, the patient should not undergo a pericardial biopsy for etiologic purposes but for therapeutic need, as Giuliani, G. et al.^3^has shown only 13.7% of all pericardial biopsies yielded a conclusive histopathological diagnosis.
